# Reducing uncertainty in simulation estimates of the surface tension through a two-scale finite-size analysis: thicker is better†

**DOI:** 10.1039/c9ra07058c

**Published:** 2019-11-04

**Authors:** José L. Rivera, Jack F. Douglas

**Affiliations:** Laboratorio de Modelamiento y Simulación Molecular, Universidad Michoacana de San Nicolás de Hidalgo Morelia Michoacán 58000 Mexico jlrivera@umich.mx; Materials Science and Engineering Division, National Institute of Standards and Technology Gaithersburg Maryland 20899 USA

## Abstract

Recent simulation studies of the surface tension *γ*, and other properties of thin free-standing films, have revealed unexpected finite size effects in which the variance of the properties vary monotonically with the in-plane width of the films, complicating the extrapolation of estimates of film properties to the thermodynamic limit. We carried out molecular dynamics simulations to determine the origin of this phenomenon, and to address the practical problem of developing a more reliable methodology for estimating *γ* in the thermodynamic limit. We find that there are two distinct finite size effects that must be addressed in a finite size analysis of *γ* in thin films. The first finite size scale is the in-plane width of the films and the second scale is the simulation cell size in the transverse direction. Increasing the first scale enhances fluctuations in *γ*, measured by the standard deviation of their distribution, while increasing the second reduces *γ* fluctuations due to a corresponding increased ‘freedom’ of the film to fluctuate out of plane. We find that using progressively large simulation cells in the transverse direction, while keeping the film width fixed to an extent in which the full bulk liquid zone is developed, allows us to obtain a smooth extrapolation to the thermodynamic limit, enabling a reduction of the *γ* uncertainty to a magnitude on the order of 1% for systems having a reasonably large size, *i.e.*, *O* (1 μm).

## Abstract 2

The variance in the surface tension of systems under vapor/liquid equilibrium is strongly affected by the size of the interfacial area. Wider layers increase the variance, but these increments disappear as the interfacial area grows.

## Graphical abstract

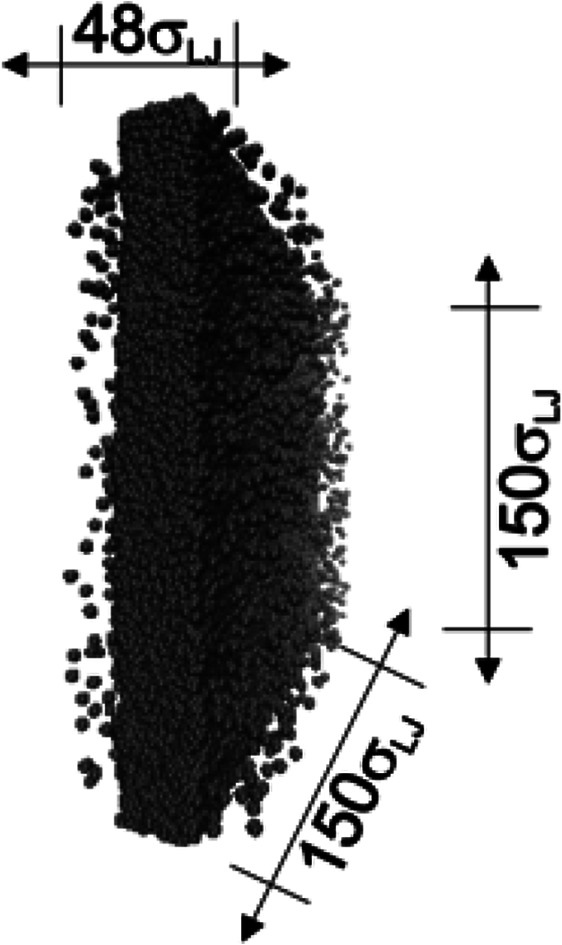

## Introduction

Longford *et al.*^1^ recently studied the vapor/liquid equilibrium of Lennard-Jones atoms (Ar) and water molecules, using thin liquid films surrounded by coexisting vapor phases, and found unexpected finite-size effects for the variance of *γ* as the width of the simulated film was varied. This result is important because it suggests that extrapolation of the film properties by increasing the film in-plane extent may not lead to reliable thermodynamic limit estimates of *γ* and other film properties. For Lennard-Jones systems with liquid and vapor lengths, and dimensions in the transverse directions of between 15*σ*_Ar–Ar_ and 35*σ*_Ar–Ar_ (*σ*_Ar–Ar_ being the diameter of a single argon atom), temperatures between 85 K and 135 K, a short reduced-cutoff radius of 
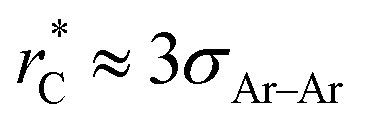
 with long-range corrections to the properties, and a large timestep of 2 fs, the variance of the surface tension grows with the length of the layers in a linear fashion. Schmitz *et al.*^2,3^ suggested that these unexpected size effects are due to a ‘domain broadening’ caused by density fluctuations in the bulk liquid. In a previous study, we found that bulk and some interfacial properties of narrow layers vary with the size of the simulation cell in the transverse directions, and that the average property values and their distribution narrow as the transverse area of the simulation cell is increased.^4^ Here, we perform simulations aimed to understand these unexpected finite size effects, also observed by Langford *et al.*,^1^ in order to develop a more reliable approach for the precise estimation of the thermodynamic properties of thin films.

In this study, we examined two different finite-size effects on the variance of *γ*, where we focused on the variance because this quantity characterizes the distributions of our *γ* estimations, which are normally distributed. First, by considering wider films composed of Lennard-Jones atoms, and longer 
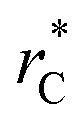
 to avoid the use of long-range potential corrections, we found a more complex behavior for the distribution of values of *γ* than those reported by Longford *et al.*,^1^ who used a small 
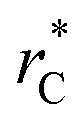
 with long-range corrections to the value of *γ*. We also found that the length of the simulation cell in the transverse direction makes the standard deviation of *γ* decrease significantly as this scale increases, an effect apparently arising from the greater freedom of the interface to fluctuate out of plane when the transverse simulation scale is larger. There is evidently a two-scale finite size phenomenon arising in simulations, which is natural given the inherently anisotropic character of fluid films. Recognition of this two scale finite size effects allows us to develop a more reliable extrapolation method based on making the transverse film thickness larger while holding the in-plane films dimensions fixed, enabling the monotone extrapolation of film properties to the thermodynamic limit.

## Methodology

In molecular dynamics simulations, the properties of fluids are commonly calculated as the mean value of data points that represent averages of blocks of timesteps calculated over up to millions of timesteps (which are on the scale of fs), with errors (standard deviation, variance, *etc.*) being calculated as a function of those average block values. However, this procedure does not provide an idea of how the film properties fluctuate as the system evolves in time. The use of block averaging adds uncertainty to the calculation of film properties because this type of averaging can hide periodic phenomena related to natural physical phenomena occurring over this averaging timescale, *e.g.*, the expansion and contraction processes in vapor/liquid equilibrium systems. The improper choose of the timescale of the block averaging can lead to inaccuracies in calculated properties.

**Fig. 1:**
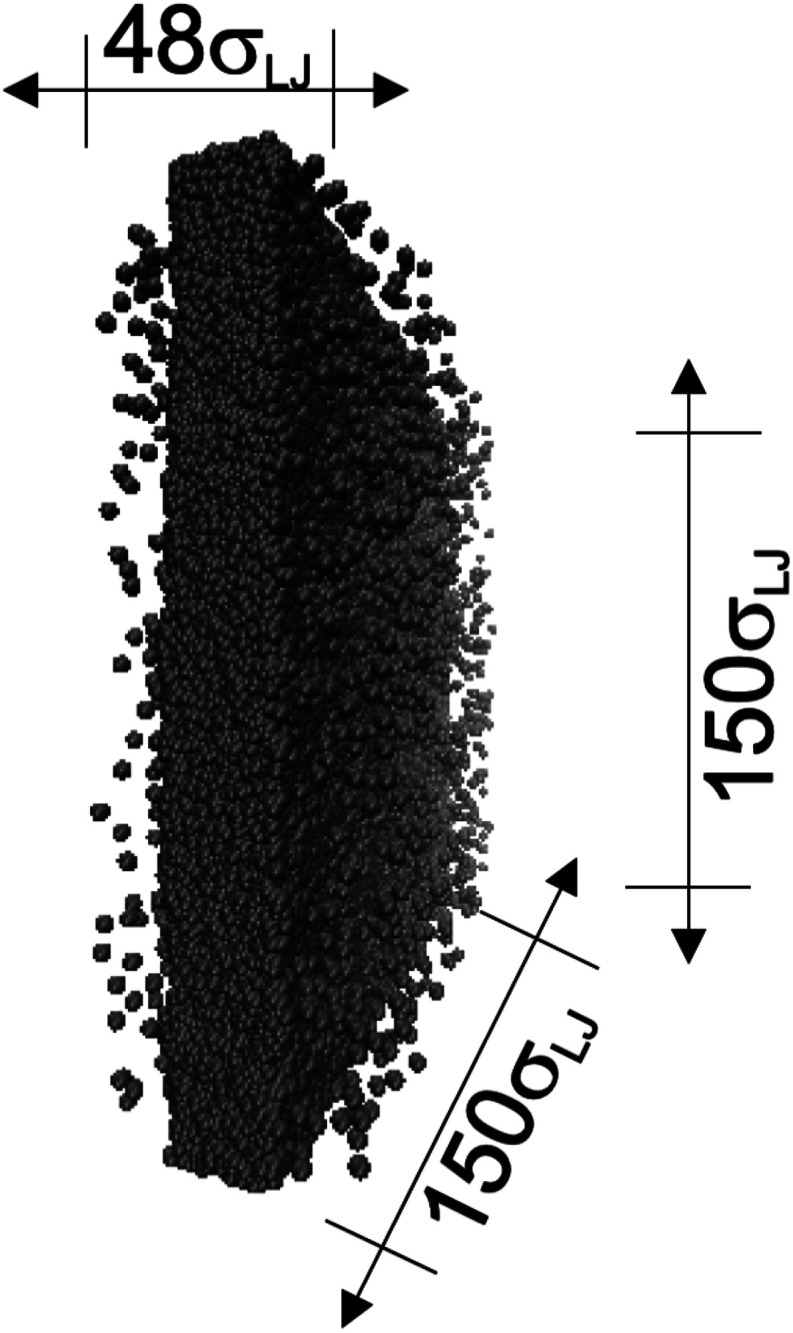
Orthogonal projection of a snapshot of the central cell used in the molecular dynamics simulations. The marked dimensions correspond to the reduced lengths for the simulation cell with the widest interfacial area studied. Each sphere represents a Lennard-Jones atom at a reduced temperature of 0.72.

The vapor/liquid thermophysical and interfacial properties of the Lennard-Jones fluid was studied using molecular dynamics simulations at a reduced temperature T* of 0.72, which is in the vicinity of the triple point of the fluid. Direct simulations of interfaces are commonly used to study the vapor/liquid equilibria of pure and multicomponent systems, including polarizable systems, formed by thin layers of liquid surrounded by a vacuum or vapor phases in thermodynamic equilibrium.^5–10^ The simulation cell consisted of a parallelepiped, with reduced dimensions of between 16 and 150.4 in the homogeneous directions (interfacial surface), and 48 in the inhomogeneous direction (which includes two interfaces), giving reduced interfacial areas between 256 and 22 620, and contained between 1040 and 336 346 Lennard-Jones atoms (Fig. 1). Because there are two interfaces in our systems, we need to simulate vapor/liquid systems with enough vapor space to be in equilibrium with the liquid central layer. Systems with a ratio of 2 between the vapor and the liquid volumes are commonly employed,^11,12^ so that liquid layers having a reduced thickness ≈ 16 requires a simulation cell having reduced length of 48 in the direction in the inhomogeneous direction. Performing the simulations near the triple point with this choice of cell anisotropy ensures that the capillary waves in the surface layer do not lead to the system becoming unstable. The initial systems consisted of solids with a face-centered cubic conformation, which were brought to the vapor/liquid equilibrium slowly, over a period of 10^6^ steps. Film properties studied were calculated using additional simulations in an NVT ensemble (constant number of molecules, volume of the rigid simulation and temperature) for a period of 10^7^ steps, with a reduced timestep of 0.005. A Nosé^13^ thermostat was used, implemented in the large-scale atomic/molecular massively parallel simulator (LAMMPS).^14^

Intermolecular interactions were calculated using the Lennard-Jones potential, which, in its reduced form, is:1
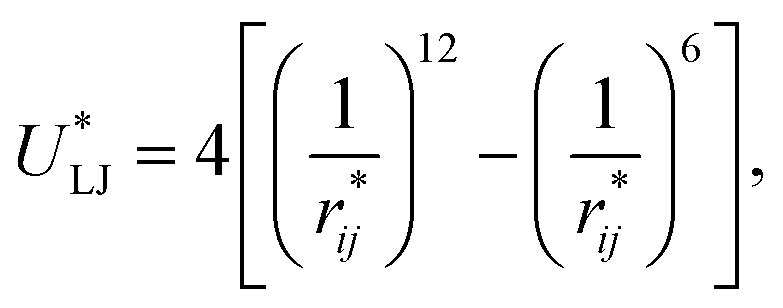
where 
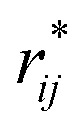
 represents the reduced separation between Lennard-Jones atoms *i* and *j*. The reduced Lennard-Jones potential was used with a 
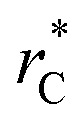
 of 7.5. By using this long 
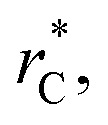
 all significant intermolecular interactions are taken into account, and the use of long-range corrections to calculate the total value of the properties is avoided.^15^ The use of a short 
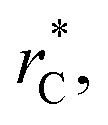
 with long-range corrections, allows for reasonable predictions of the average vapor/liquid equilibrium properties, but the dynamics of the system are dependent on the 
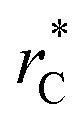
 employed. In this study, we used reduced variables and properties, which for fluids governed by the Lennard-Jones potential is an effective way of studying the corresponding states of Lennard-Jones fluids. The properties of fluids in laboratory units can be calculated from the reduced properties (indicated by the addition of an asterisk) using the *σ* and *ε* parameters of the Lennard-Jones potential associated with the specific fluid studied. Real lengths are calculated as *l* = l*σ, time *t* = t*(*mσ*^2^/*ε*)^1/2^, energy *U* = U*ε, force *F* = F*ε/*σ*, temperature *T* = T*ε/*k*_B_, density *ρ* = ρ*/*σ*^3^, pressure *P* = P*ε/*σ*^3^ and surface tension *γ* = γ*ε/*σ*^2^ and *k*_B_ is Boltzmann's constant.

The pressure profiles were obtained through the calculation of pressure tensors in each slab, using Harasima pressure profiles,^16,17^ implemented in LAMMPS,^18^ in which the contributions to the profiles are distributed only in the two slabs that originated the interactions, and not evenly distributed through the slabs that lie between those that originated the interactions.^9,19^ The normal pressure profiles obtained from this definition vary along the interface – a trend often dismissed as being unphysical, although it is not feasible measure these predicted profiles from the components of the pressure in the interfacial region. Even so, the pressure profiles of Harasima have been used in several surface-tension studies because it does not matter whether the interaction forces are distributed uniformly in several slabs, or in only two; when it is integrated to obtain the profile of the surface tension, the same result is obtained.^10,20–23^ As the contributions to the Harasima pressure profiles are located in the regions that originated the interactions, the density inhomogeneities at the interfaces are highlighted in the Harasima profiles. The Harasima normal pressure profiles are mechanically stable because the outermost regions show large negative pressures (attractive) holding together the whole liquid layer. Before the attractive zones, there are small peaks with positive pressure (repulsive), which, in comparison to the attractive ones, are smaller, and are not long and wide enough to burst the liquid layer. Here, the reduced surface tension γ* was calculated through its mechanical definition using the Harasima pressure profiles.^4,10^ In this way, the whole system (two interfaces) is used to calculate de surface tension.

In order to obtain more realistic profiles, we allowed the system to move in the inhomogeneous direction, and calculated the density and pressures profiles every 100 steps. At the end, we averaged these by correcting the positions of the profiles according to their positions in the inhomogeneous direction of the center of the layer, which was calculated as the midpoint between the two positions of the Gibbs' dividing surface of each interface, 
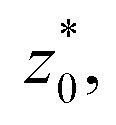
 with the thickness of the layer, 
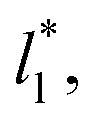
 corresponding to the separation between the positions of the two dividing surfaces. This allowed us to obtain more realistic profiles of the calculated properties. To obtain the positions of 
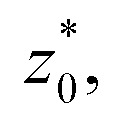
 each of the density profiles, calculated every 100 steps, was adjusted to the hyperbolic tangent expression commonly used in vapor/liquid phase equilibrium studies:^11,19^2
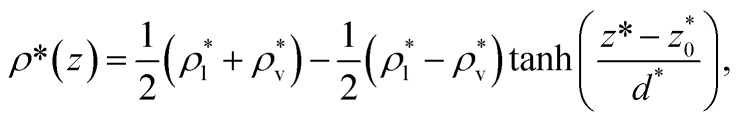
where 
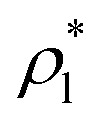
 and 
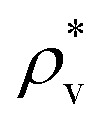
 are the average bulk densities of the liquid and vapor phases, respectively, and d* is a measure of the thickness of the interface, and describes the length in the inhomogeneous direction, where the density changes from the bulk liquid to the bulk vapor phase.

## Results

Molecular dynamics are commonly carried out in finite simulation cells, and it is important to consider those finite effects of the simulation cell on the property calculations.^24–26^ Two finite-size variables that can be studied are the length of the layer and the interfacial area. Lengths of the layer below a critical thickness induce a layer break-up, while at the critical thickness, they produce metastable systems with no real bulk liquid phase.^4^ Layers that are wide enough correspond to coexisting phases that are thermodynamically stable, and it is expected that further increments in the thickness will not change the interfacial and coexisting average properties. The size effects in the interfacial area are not intuitive due to the periodic boundary conditions employed, which try to reproduce the dynamics of systems with infinite and continuous interfacial areas; however, the periodic images create artificial cohesive forces at the interfaces, affecting certain thermophysical and interfacial properties, while the average surface tension remains almost insensitive.^4^ The cohesive forces are stronger in simulation cells having smaller interfacial areas, and result of the localization of the Lennard-Jones atoms, and thus enhance the interatomic cohesive interactions, in the interfacial region. Due to the cutoff of the Lennard-Jones potential used, we performed the simulations using a reduced interfacial length, 
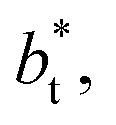
 from 16 to 150.4, and thicknesses from the critical length to a reduced length of 16.8. Also, for the system with the smallest interfacial area used, we simulated systems with reduced lengths of up to 67.8.

**Fig. 2:**
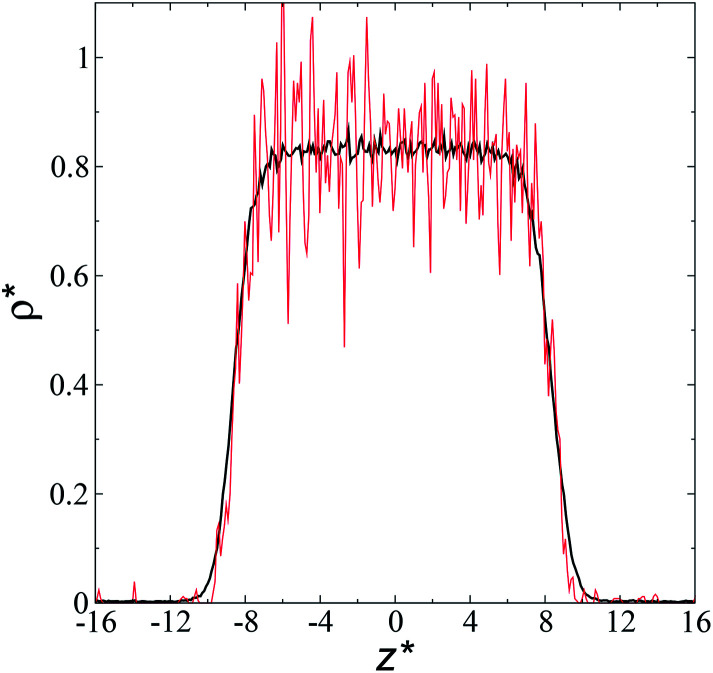
Reduced density profiles as a function of the position in the inhomogeneous direction of the simulation cell. The profiles represent average profiles of 100 timesteps. The systems are composed of Lennard-Jones atoms at a reduced temperature of 0.72 and reduced thicknesses of ≈16.8. Red and black lines correspond to simulated systems using reduced lengths of the simulation box in the tangential direction of 16 and 150.4, respectively.

**Fig. 3:**
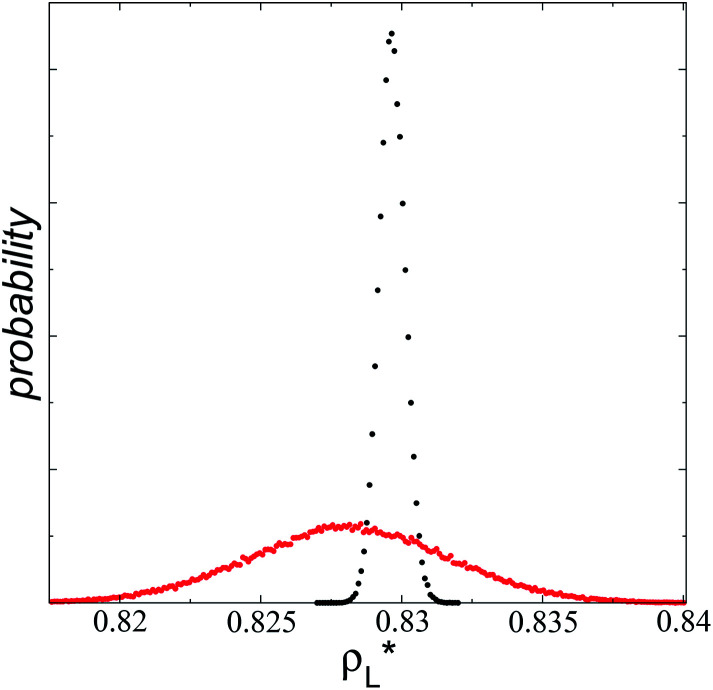
Histograms of the liquid densities averaged over 100 timesteps. The systems are composed of Lennard-Jones atoms at a reduced temperature of 0.72 and reduced layer thicknesses of ≈16.8. Red and black profiles correspond to simulated systems using reduced lengths of the simulation box in the tangential direction of 16 and 150.4, respectively.

The artificial cohesive forces arising from the periodic boundary conditions induce a more structured behavior of the systems in the bulk phases and at the interfaces. Fig. 2 shows the density profiles averaged over 100 timesteps for systems with 
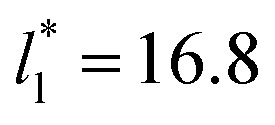
 (far away from the critical length^4^), T* = 0.72 and 
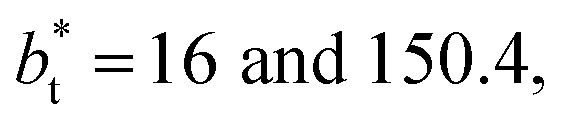
 which represent the smallest and largest surface areas studied. Even these profiles can be more well defined using more timesteps to average the profiles, the average over 100 timesteps allow us to understand the instantaneous density profile estimates. The system with a 
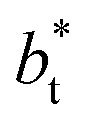
 of 16 was more affected by the finite size area and produced larger fluctuations of the density not only in the bulk liquid, but also in the vapor phase and the interfaces. Density profiles averaged over 10^6^ timesteps or more produced smooth, and almost identical, density profiles, with very small difference in the average values in the bulk phases 
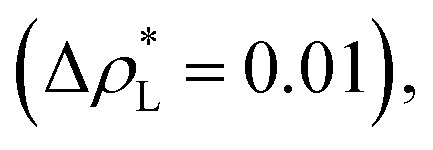
 and equal to those previously reported from studies of phase equilibria, and obtained by Trokhymchuk and Alejandre using a 
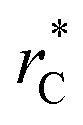
 of 5.5.^15^ Even the average densities are almost equal for both 
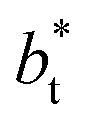
 values, the distributions of instantaneous bulk liquid densities are different. Using 
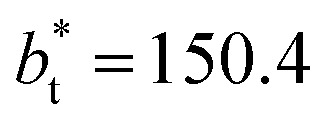
 produced a very narrow distribution of instantaneous bulk liquid densities when compared with the distribution at 
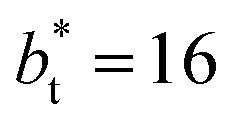
 (Fig. 3). Similar behavior has been observed for distributions of the surface tension,^4^ where the wide distributions are attributed to perturbations created by the induced cohesive forces, which are larger when small interfacial areas are used in the simulation cells. Comparing sections of 
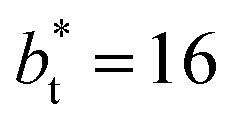
 in the larger system with respect to the smaller system 
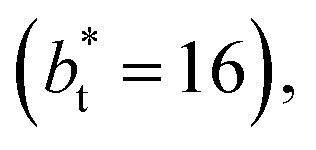
 we can expect that the interfacial sections of size 
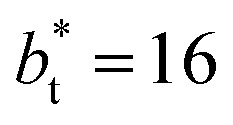
 in the larger system will have as much noise as the section of the smaller system, but these sections will interact collectively producing configurations with some sections having small surface tension, while in the other sections a relatively large surface tension is found due to out-of-plane expansions and contractions of the film. These fluctuations narrow the distribution of values of the surface tension and liquid density in larger systems.

**Fig. 4:**
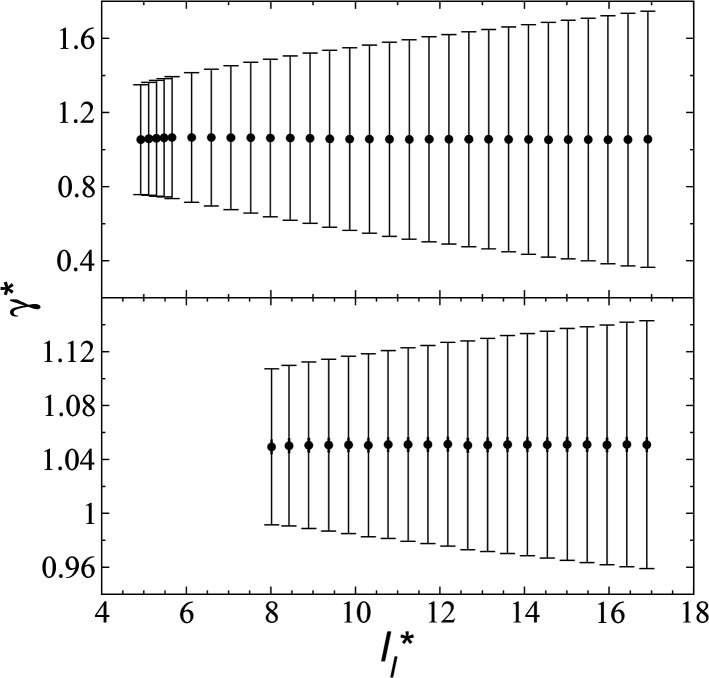
Surface tension as a function of the length of the layer for simulations cells with reduced tangential lengths of 16 (top) and 120 (bottom). The systems are composed of Lennard-Jones atoms at a reduced temperature of 0.72. The shortest lengths correspond to layers at their critical lengths.^4^ The error bars correspond to their standard deviations.

In a similar way, the average γ* is also insensitive to the value of 
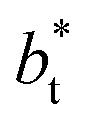
 employed in the simulation, as previously reported,^4^ but also it is insensitive to the length of the layer, as shown in Fig. 4. At constant 
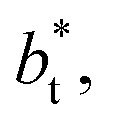
 the average values of γ* remain insensitive as 
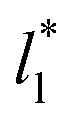
 grows, while the corresponding standard deviation grows. For layers with 
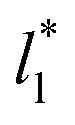
 between 4.3 and 40.0, Werth *et al.*^27^ found an inverse cubic dependency between γ* and 
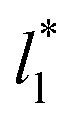
 using a 
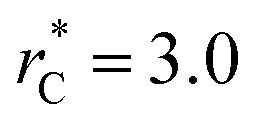
 and long-ranged corrections to γ*, but this dependency is only evident in very narrow layers 
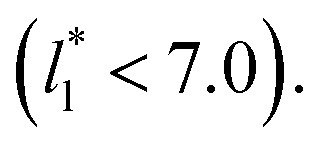
 For layers with 
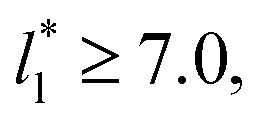
 corresponding to range investigated in the present work, the reported surface tension values at the lower T* of 0.70 are very similar, *i.e.*, 1.13 ± 0.03 for 
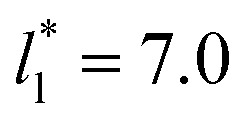
 and 1.150 ± 0.004 for 
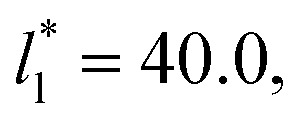
 which accords with our finding that γ* is insensitive to 
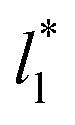
 over the range of T* and 
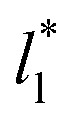
 investigated. Moreover, the standard deviation values of γ* did not reduce its magnitude when they were calculated over longer periods of simulation, probably indicating that smaller standard deviations are only produced when standard deviations are calculated using average values of γ* of time-blocks, probably hiding periodic phenomena. When comparing the average values of the two sets at different 
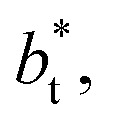
 for each 
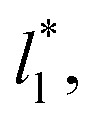
 we found very small differences that were less than the size of the symbols. On the other hand, we found a similar behavior between the distributions of the bulk liquid density and the distributions of the surface tension, in which the distributions of γ* narrowed as 
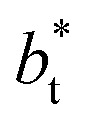
 increased, which manifested as lower standard deviations. We also note that Chen^28^ reported an inverse quadratic dependency of γ* on 
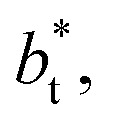
 but their reported estimates of γ* are quite small, and were based on simulations utilizing a relatively small number of atoms (≤4080), the introduction of a small cut-off 
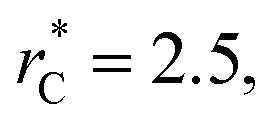
 no long-ranged corrections to γ*, and 
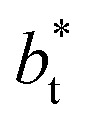
 values significantly smaller than those used in the present work 
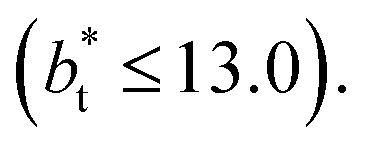
 Given these differences in the model calculation assumptions of Chen and our own, a direct comparison with our results is not possible. We note that an inverse quadratic dependency of γ* on 
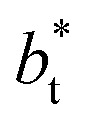
 cannot be ruled out for large 
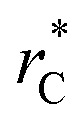
 in this range of 
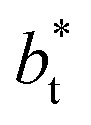
 values because of the presumably metastable nature of the film states under these conditions.^4^ We infer from Chen and our own results that future work should systematically investigate the role of the choice of cut-off on film property estimates since this would appear to be another source of uncertainty in molecular dynamics simulation estimation of film properties.

**Fig. 5:**
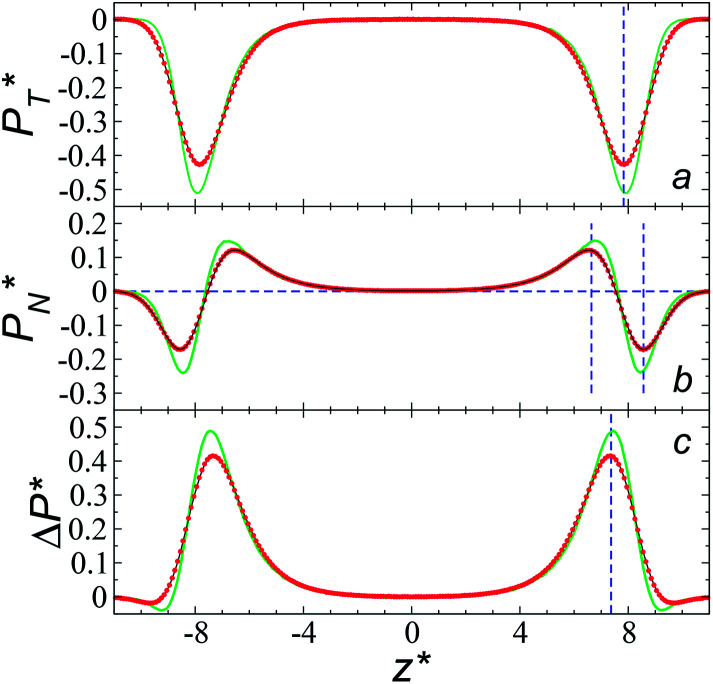
Average tangential (a) and normal (b) pressure profiles, and the differences between them (c). The systems are composed of Lennard-Jones atoms at a reduced temperature of 0.72 and reduced thicknesses of ≈16.8. Green, black and red lines correspond to simulated systems using reduced lengths of the simulation box in the tangential direction of 16, 140.8 and 150.4, respectively. Blue lines represent the positions of peaks in the profiles corresponding to the simulated system, using a reduced length of the simulation box in the tangential direction of 150.4.

We studied the origins of the insensitivity of the behavior of the average value of γ* with the size of 
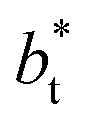
 at constant 
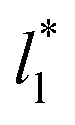
 through analyzing the components of the average pressure profiles. In Fig. 5, we plotted the normal 
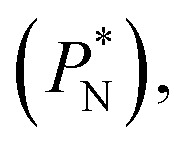
 transverse 
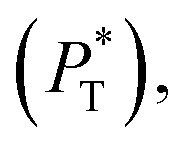
 and difference between the average normal and average tangential pressure profiles 
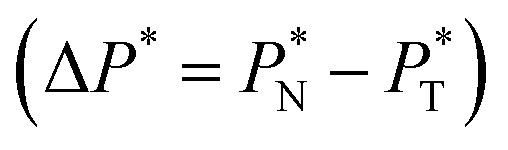
 for systems with 
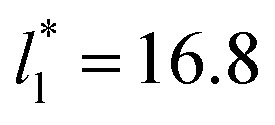
 and 
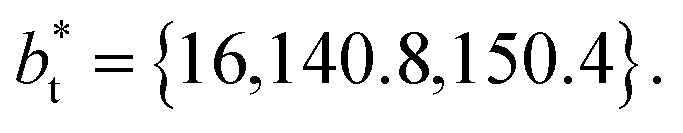
 The 
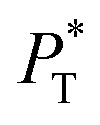
 profiles show the regular behavior of near-zero values in the bulk zones and large negative peaks at the interfaces, which are the result of the inhomogeneous density at the interface. At the interfacial zones, the average 
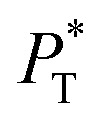
 profiles show clear differences between the system simulated using 
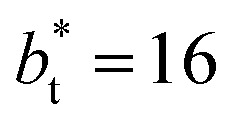
 and the systems simulated at larger 
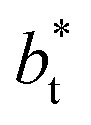
 sizes. In the reduced units, the minimum in the negative peak for the system simulated using 
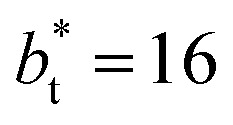
 is ≈0.09 deeper than the systems simulated using larger 
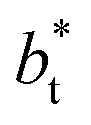
 sizes, while at the outermost part of the interface, the profile using 
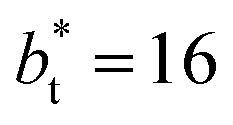
 shows slightly lower values. The systems simulated at 
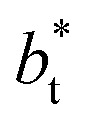
 sizes of 140.8 and 150.4 are identical, and reflect the visual disappearance of the size effects on the average 
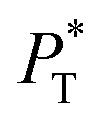
 profiles using large 
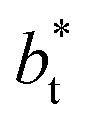
 sizes.

**Fig. 6:**
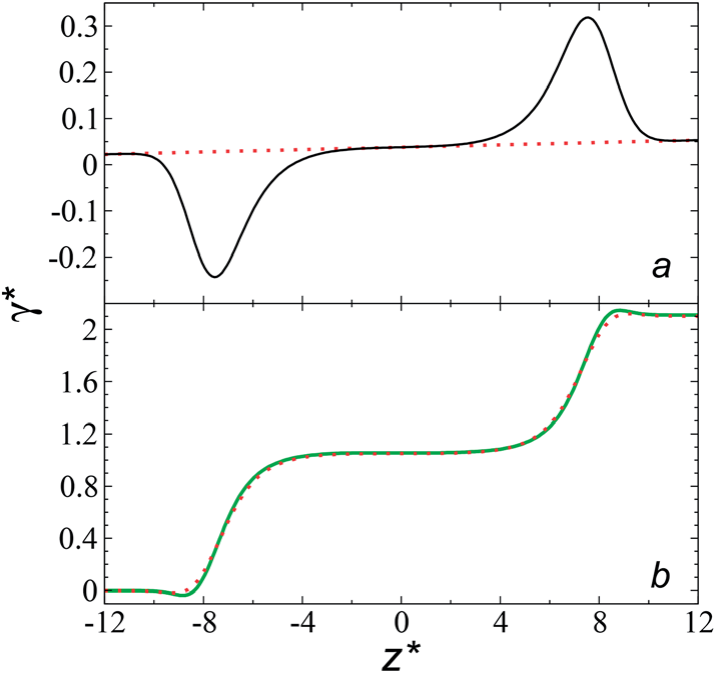
(a) Integration of the average normal pressure profile as a function of the reduced position in the simulation cell (black line). The systems are composed of Lennard-Jones atoms at a reduced temperature of 0.72, using a reduced length of the simulation box in the tangential direction of 150.4, and reduced thicknesses of 16.8. The red line corresponds to the integration of a profile with a constant normal pressure along the bulk phases and interfaces. (b) Surface tension profiles as a function of the reduced position in the simulation cell. Green and red lines correspond to simulated systems using reduced lengths of the simulation box in the tangential direction of 16 and 150.4, respectively.

The average 
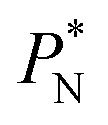
 profiles also show the regular behavior of the Harasima pressure profiles, which are not flat at the interface. They depict a more intuitive picture, wherein the outermost monolayer of atoms is strongly attracted by the bulk liquid, originating the outermost negative peak (cohesive) on the average 
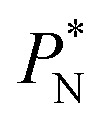
 profiles. The strongly attracted outermost monolayers crunch a monolayer of atoms located between them and the bulk liquid, which traduces in the positive peaks (repulsive) located in the reduced positions of ±7.5 
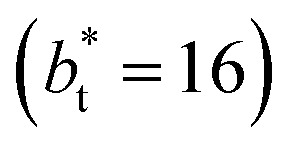
 and ±7.7 
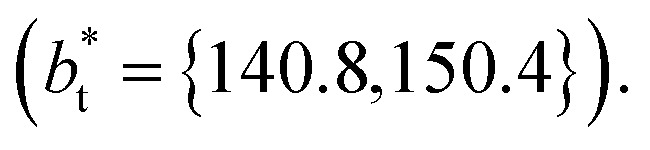
 The average 
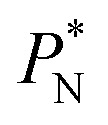
 profiles also show differences depending on the size of 
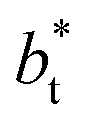
 employed during the simulation. The maxima and minima in the peaks for the system simulated using 
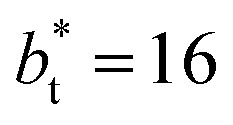
 are larger by ≈0.03 (positive peaks) and ≈−0.06 (negative peaks) reduced pressure units than the systems using larger 
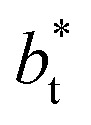
 sizes. Close to the outermost part of the interface, the average 
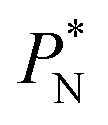
 profiles, using 
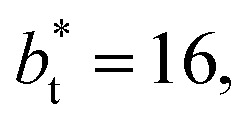
 also show lower values than the profiles using larger 
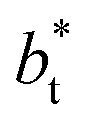
 values, which can affect the processes of vaporization/condensation, which were beyond the scope of this study. The integration of the average Harasima 
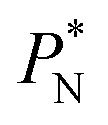
 profiles results in a net contribution identical to the integration of a flat 
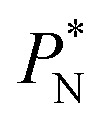
 profile, which some authors have assumed has a physical sense (Fig. 6a). The slope of the net contribution is not zero because the average 
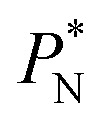
 in the bulk zones is not zero, instead it corresponds to the saturation pressure.

The average ΔP* profiles also show a regular behavior, with large positive peaks located at the interfaces and small negative peaks located in the outermost parts of the interface. The positions of the large positive peaks are identical for small and large sizes of 
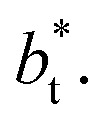
 The maxima and minima in the peaks for the system simulated using 
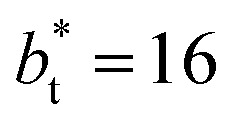
 are larger by ≈0.07 (positive) and ≈–0.02 (negative) reduced pressure units than the systems using large 
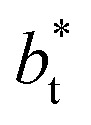
 sizes. The negative minimum for the system simulated using 
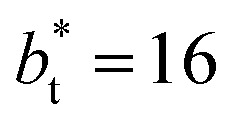
 is located at 0.5 reduced length units off the position of the systems using larger 
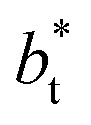
 sizes. When we integrated these profiles, we obtained the γ* profiles (Fig. 6b). Even the ΔP* profiles show small differences depending on the 
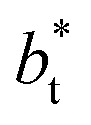
 value employed, with the γ* profiles are identical. Therefore, we can attribute the insensitivity of γ* to 
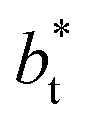
 as the result of a balance between the positive contributions of 
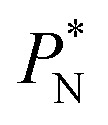
 (positive peak) and 
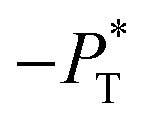
 and the negative contribution of 
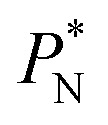
 (negative peak). For the simulated system using 
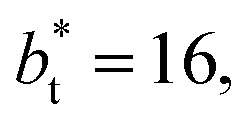
 the area of the positive peak in the average ΔP* profiles indicate a larger surface tension contribution than the peaks obtained using larger 
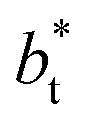
 sizes, but the outermost negative contributions are also larger for 
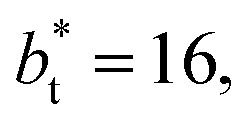
 which match the larger positive contributions, resulting in an annulment of the additional contributions.

**Fig. 7:**
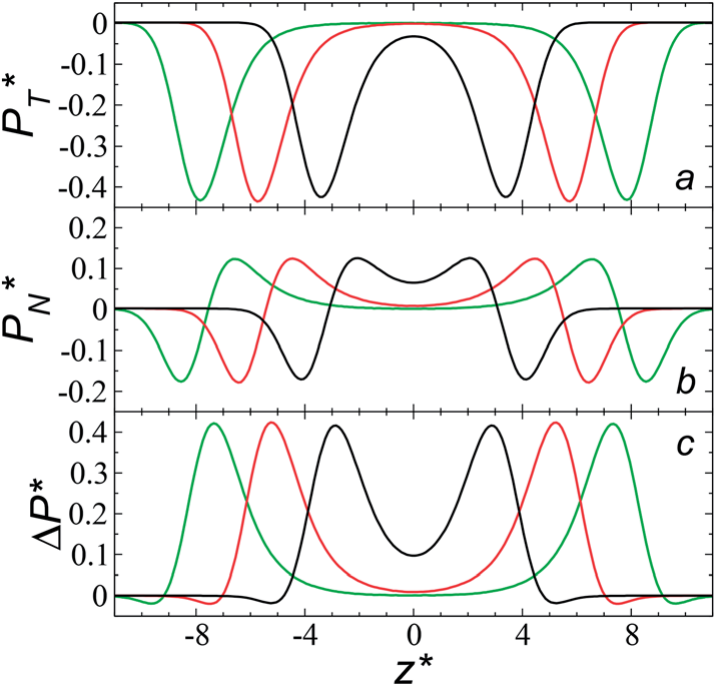
Average tangential (a) and normal (b) pressure profiles, and the differences between these (c). The systems are composed of Lennard-Jones atoms at a reduced temperature of 0.72. Profiles were calculated using a reduced length of the simulation box in the tangential direction of 120. Black, red and green lines correspond to simulated systems with lengths of the layer of 8.0, 12.6 and 16.8, respectively.

**Fig. 8:**
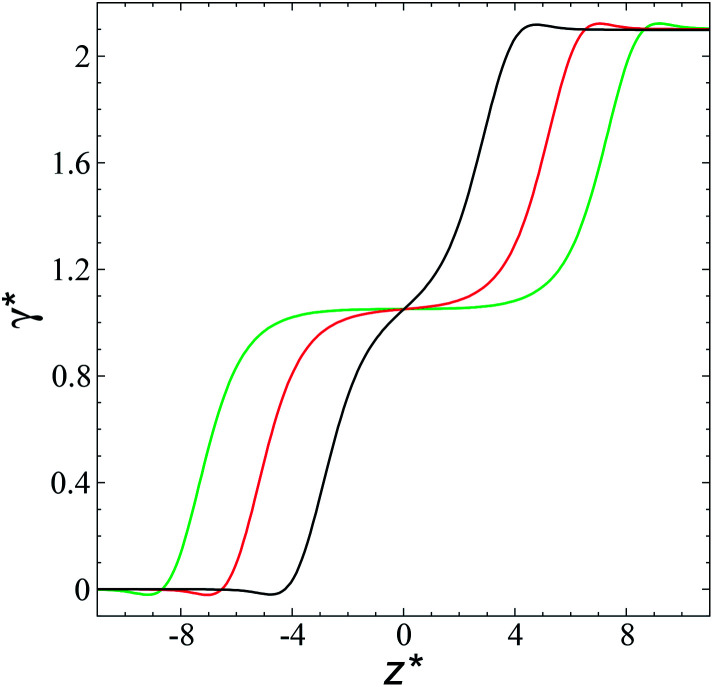
Surface tension profiles as a function of the reduced position in the simulation cell. The systems are composed of Lennard-Jones atoms at a reduced temperature of 0.72. Profiles were calculated using a reduced length of the simulation box in the tangential direction of 120. Black, red and green lines correspond to simulated systems with lengths of the layers of 8.0, 12.6 and 16.8, respectively.

The study of the pressure profiles at constant 
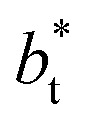
 allowed us to examine the insensitivity of the behavior of the average value of γ* with the size of 
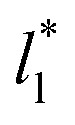
 (Fig. 7). The plotted 
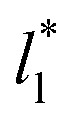
 values corresponds to 
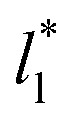
 of 8.0, 12.6 and 16.8 at a constant 
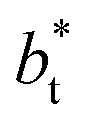
 of 120. The systems simulated using short 
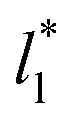
 sizes (8.0) did not develop a real bulk liquid phase in which the values of the pressure profiles reached the same values as the bulk vapor phase. This system seems to be composed of two joined interfaces. As the size of 
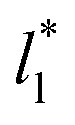
 grows, the bulk liquid zone develops, and the pressures in all profiles reach values similar to those in the bulk vapor phases. The magnitudes for all peaks for all 
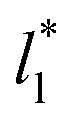
 are identical in the three pressure profiles (
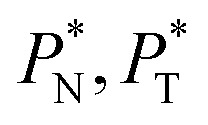
 and ΔP*). When the ΔP* profiles are integrated to obtain the γ* profiles of the two interfaces (Fig. 8), all profiles with different 
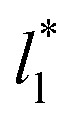
 produce the same average magnitude of γ*. The profiles of γ* for systems using larger 
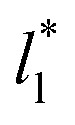
 show a central flat region, which corresponds to the bulk liquid zone, which reduces until it disappears at the shortest size of 
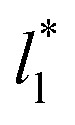
 reported. Even the ΔP* profiles using short sizes for 
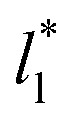
 do not reach equilibrium values when they are integrated to produce the γ* profiles; instead, they produce the same average γ* calculated using larger 
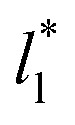
 sizes (Fig. 8). As 
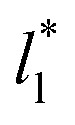
 decreases, the interfaces seem to join (Fig. 7), producing a central zone characterized by large normal (positive) and tangential (negative) pressures. As the interfaces join, the pressures of the interfaces, which give rise to the surface tension, do not cancel out or disappear, rather they accumulate in the short central zone, ultimately producing the same average value for the surface tension due to the symmetry of the two interfaces in the ΔP* profile. Werth *et al.*^27^ previously reported the same dependency of the ΔP* profiles with 
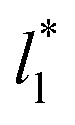
 at the lower T* = 0.70, which confirms the equivalency between the Harasima pressure profiles^16,17^ used in this work and those produced with the homogeneously distributed pressures of Irving and Kirkwood.^29^

**Fig. 9:**
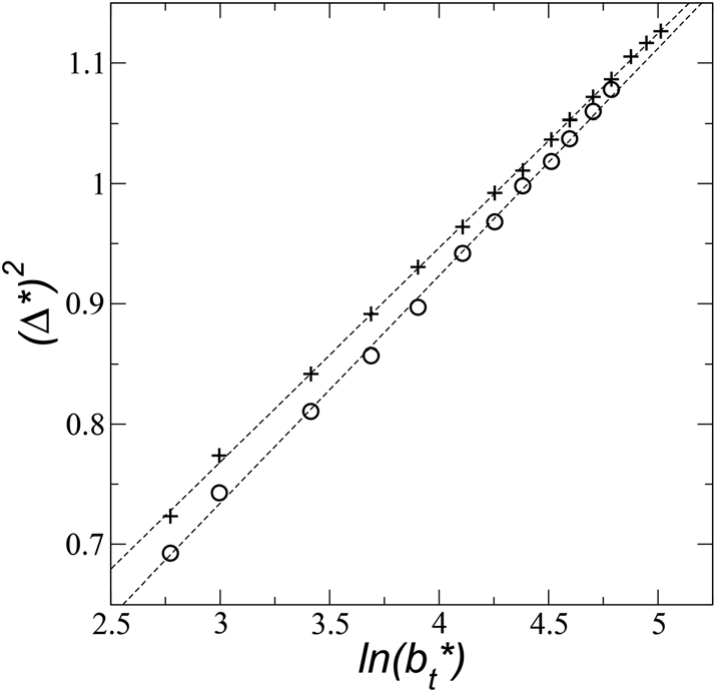
Square of the reduced interfacial thickness as a function of the length of the simulation cell in the tangential direction. The systems are composed of Lennard-Jones atoms at a reduced temperature of 0.72. Circles and crosses correspond to layers with reduced thicknesses of 8 and 16.8, respectively. Discontinuous lines correspond to linear regressions.

An insensitivity of the average γ* to 
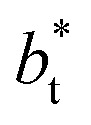
 is expected in capillary wave theory,^30^ in which the interfacial thickness, *Δ*, can be decoupled into an intrinsic contribution, *Δ*_0_, and a logarithmic contribution. The interfacial thickness depends on the size of the simulation cell in the transverse direction (which directly correlates with the size of the capillary waves that can form at the interface), while the average *γ* remains constant. Larger simulation cells induce wider capillary waves, making the interfacial thickness also wider (at very large 
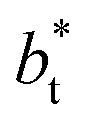
 values, probably unrestricted). In laboratory units:3
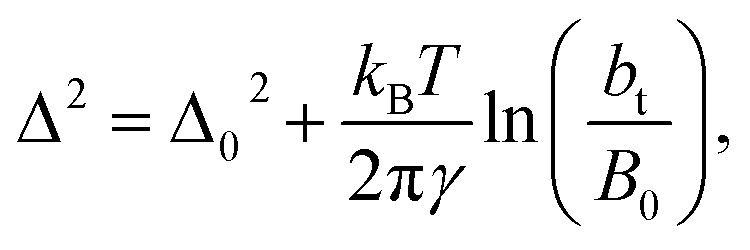
where *B*_0_ is a characteristic size of the simulation cell in the transverse direction. At very short *b*_t_ sizes (close to *B*_0_), the interfacial thickness is equal to their intrinsic value. A study on water simulations have employed eqn (3) to predict the surface tension at a specific thickness of the layer,^30^ but no comparisons have been made at different thicknesses of the layer, and none have explored the limits of eqn (3) at very narrow thicknesses. Fig. 9 shows (Δ*)^2^ as a function of 
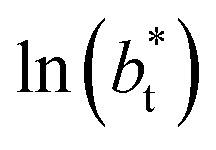
 for the systems with 
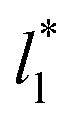
 of 8.0 and 16.8. Linear regressions of the two sets of data produce almost parallel lines, which also reflect the insensitive behavior of γ* not only with 
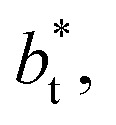
 but also with 
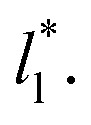


**Fig. 10:**
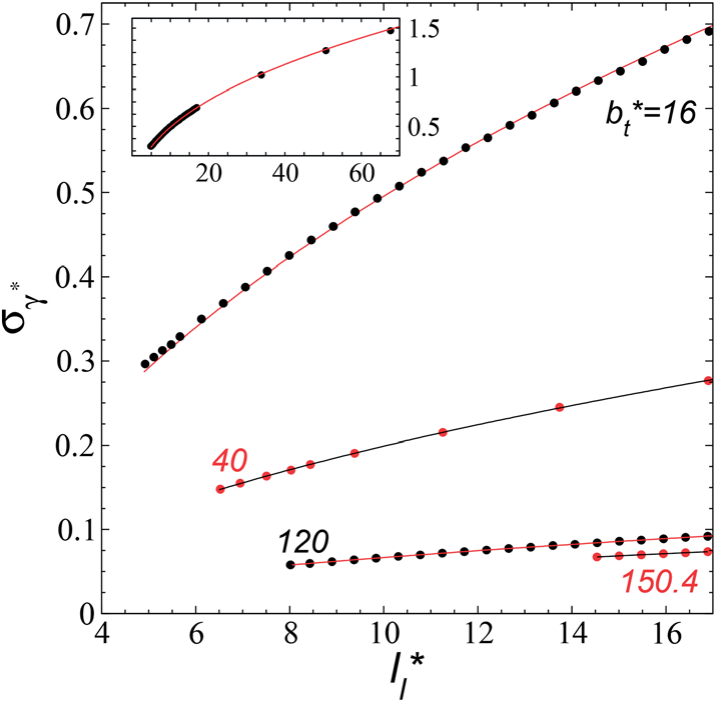
Standard deviation of the reduced surface tension as a function of the reduced length of the liquid layer of Lennard-Jones atoms. The reduced temperature of the systems is 0.72. The four sets of data represent four simulation cells with reduced lengths in the tangential direction of 16, 40, 120 and 150.4. The lines for the first three systems represent the best fits to eqn (4), while the line for the largest simulation cell size represents a linear regression. The inner graph shows the extended data for the smallest simulation cell size studied (16) and the best fit to eqn (4) at lower values. The axis labels are the same for both graphs.

The standard deviation of γ*, *σ*_γ*_, is plotted as a function of 
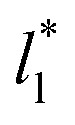
 in Fig. 10 for the simulated systems using sizes of 
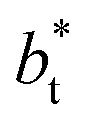
 of 16, 40, 120 and 150.4. We calculated the profiles of *σ*_γ*_ for each 
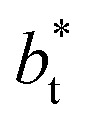
 from the first stable layers at its critical length,^4^ with thicknesses corresponding to a few monolayers, up to a reduced value of 
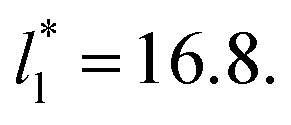
 The profiles show increasingly smaller values of *σ*_γ*_ as 
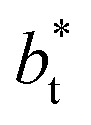
 increases. The inner graph of Fig. 10 shows additional data results for the smallest size of 
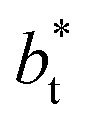
 used (16) for thin layers with 
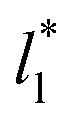
 up to 67.8; the profiles of *σ*_γ*_ seem to follow a power function behavior with 
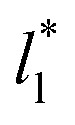
. The profiles of the simulated systems 
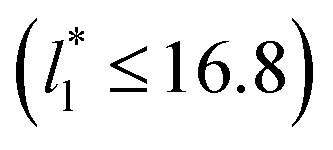
 using larger values of 
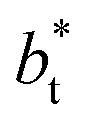
 seem to follow a near-linear behavior with 
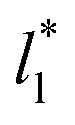
 as 
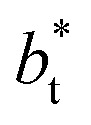
 grows. The increasingly linear behavior of the profiles with 
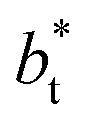
 can be modeled through a power function of the form:4
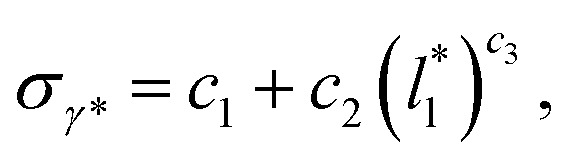
which produces values for the *c*_3_ power exponent of 0.43, 0.44 and 0.49 for the systems with 
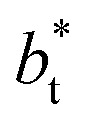
 of 16, 40 and 120, respectively. For the thin layer using 
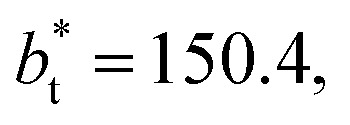
 a linear behavior is the best fit. For the systems simulated using the smallest size of 
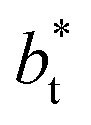
 (16), the parameters of eqn (4) fitted with thinner layers 
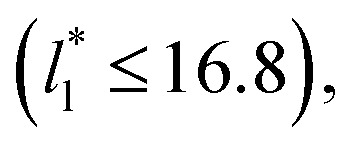
 produced values close to those of the simulation results for the wider layers 
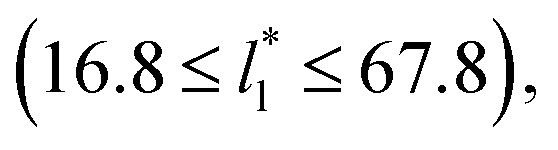
 and are also shown in the inner plot of Fig. 10, which reflects the consistency of the obtained parameters using data from the smaller 
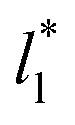
 systems. We note that our *σ*_γ*_ with 
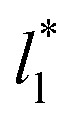
 estimates do not follow the root square dependence with system size. This unexpected behavior is because the interfacial forces vary with 
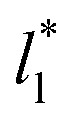
 in thin-layer systems, as can be seen in Fig. 7.

Yoon *et al.*^31^ have proposed that only a few layers of the interface should be used to calculate the surface tension to avoid the size effects of the bulk phases (length of the layer). Yoon *et al.*^31^ employed this methodology to calculate the surface tension of water using a region of ≈ 5 Å (which is less than the diameter of 2 water monolayers) in a small system composed of 804 water molecules. The limits of this region corresponded to 0.01 and 0.90 g cm^−3^, which are asymmetrical in the density profile of water, and do not correspond to the values of the so-called “10–90” thicknesses, which corresponds to limiting points in the density profile at 10% and 90% of 
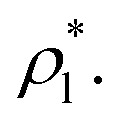
 The final result of Yoon *et al.*^31^ calculations did not produce a consistent tendency in the surface tension of water as a function of temperature. We employed this strategy to calculate the surface tension of the Lennard-Jones fluid by eliminating regions of the bulk phases, and the results are summarized in Table 1. If we eliminate parts of the bulk liquid from the integral to compute γ*, γ* and *σ*_γ*_ decreased, reaching a minimum when we eliminated a region that represents ≈70% of the layer thickness, beyond this point the behavior is ill-defined. The decrements in γ* were then less pronounced than the decrements in *σ*_γ*_, but still the maximum reduction of *σ*_γ*_ was half its original value, produces considerable changes in γ* (≈−20%). If we eliminate parts of the bulk vapor from the integral to compute γ*, the resulting changes in γ* and *σ*_γ*_ are minimal even when we eliminate a region limited by a point located 0.5 reduced units away from the Gibbs' dividing surfaces. Eliminating at the same time parts of both bulk phases as proposed by Yoon *et al.*^31^ does not produce a zero change in γ* unless you eliminate a very small region in the bulk liquid, but those changes will not reduce considerably *σ*_γ*_. These results indicate that the integration over the bulk vapor region does not contribute significantly to the average value of γ*, neither *σ*_γ*_; the main contribution comes from the region between the interface and a point located between 6.5 and 8.5 reduced units (monolayers) away from the interface, in this zone the liquid density and pressures are uniform and the pressures are equal (bulk). For these narrow layers (nanometer scales), the distributions of values of γ* in fact depend on the size of the bulk liquid, no from both bulk phases, because the contractions/expansions that occur at the interface have to be the result of expansions/contractions in the bulk liquid phase, which ultimately indicates that interfaces of narrow layers do not expand/contract independently of the size of the layer.

**Table 1:** Simulation results of the surface tension and its standard deviation for the system of Lennard-Jones atoms using a 
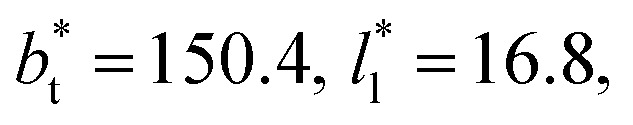
 and T* = 0.72. The integration to obtain the surface tension was carried eliminating the indicated regions of the bulk liquid, or the bulk vapor phases. The case when the whole system is integrated is presented for comparison. The whole system covers a region in reduced units of (−24 to 24), the bulk liquid is located at the center, and the Gibbs' dividing surfaces located at positions in reduced units of −8.4 and 8.4

Eliminated region (positions in reduced units)	Eliminated thickness (reduced units)	γ* (% change)	*σ*_γ*_ (% change)
	0	1.051 (0)	7.345 × 10^−2^ (0)
Liquid, (−1 to 1)	2	1.050 (−0.09)	6.819 × 10^−2^ (−7.08)
Liquid, (−2 to 2)	4	1.048 (−0.28)	6.241 × 10^−2^ (−15.03)
Liquid, (−3 to 3)	6	1.041 (−0.95)	5.602 × 10^−2^ (−23.73)
Liquid, (−4 to 4)	8	1.023 (−2.66)	4.905 × 10^−2^ (−33.22)
Liquid, (−5 to 5)	10	0.974 (−7.32)	4.179 × 10^−2^ (−43.10)
Liquid, (−6 to 6)	12	0.850 (−19.12)	3.777 × 10^−2^ (−48.57)
Liquid, (−7 to 7)	14	0.566 (−46.14)	4.464 × 10^−2^ (−39.22)
Liquid, (−8 to 8)	16	0.172 (−83.63)	3.726 × 10^−2^ (−49.27)
Vapor, (−24 to −11) ∪ (11 to 24)	26	1.052 (0.09)	7.348 × 10^−2^ (0.04)
Vapor, (−24 to −10) ∪ (10 to 24)	28	1.059 (0.76)	7.361 × 10^−2^ (0.21)
Vapor, (−24 to −9) ∪ (9 to 24)	30	1.059 (0.76)	7.407 × 10^−2^ (0.84)

We then studied the possibility that the large ratio between the tangential length and the normal length of the simulation cells used in this work can create an artefact on the calculated properties and standard deviations. For the largest system studied, which used a 
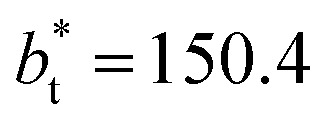
 and 
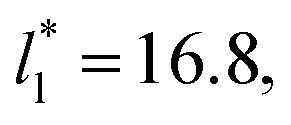
 having originally a ratio of 3.12, we simulated systems with dimensions of 96, 192 and 288 reduced length units in the normal direction, which had ratios of 1.56, 0.78 and 0.52, respectively (ESI, Fig. S1†). This additional space was spontaneously filled by vapor, and more molecules were added to maintain the 
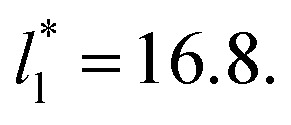
 After this procedure, we did not observe any change in the average density profiles or the average pressure profiles (see Fig. S2 and S3 of ESI†). The average values of γ* and *σ*_γ*_ are thus not affected by the aspect ratio between the cell dimensions in the tangential and normal directions, having a ratio greater than 2^1/2^ between the volumes of the vapor and liquid phase is enough. These observations are in concordance with our previous assessment of the methodology of bulk phase elimination proposed by Yoon *et al.*^31^

**Fig. 11:**
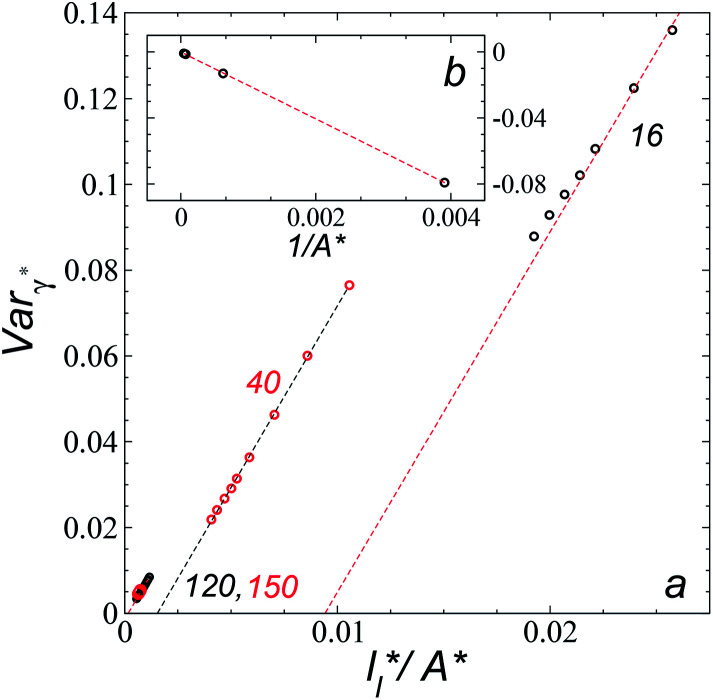
(a) Variance of the reduced surface tension as a function of the ratio of the reduced length of the layer to the reduced interfacial area of the simulation cell as proposed by Longford *et al.*^1^ Results are for reduced lengths of the simulation cell in the tangential direction of 16, 40, 120 and 150.4. The reduced temperature of the systems is 0.72. (b) Intercepts of the variance of the reduced surface tension (a) with the *y*-axis as a function of the interfacial area of the simulation cell. Discontinuous lines represent the best linear regression of each set of points.

Longford *et al.*^1^ developed an expression showing the dependency of the variance of γ*, Var_γ*_, which is the square of *σ*_γ*_, with respect to a variable that combines the two finite size effects, 
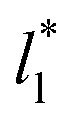
 and the interfacial area of the simulation cell, 
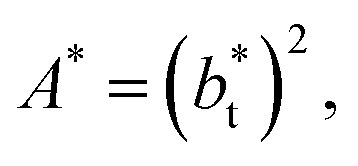
5
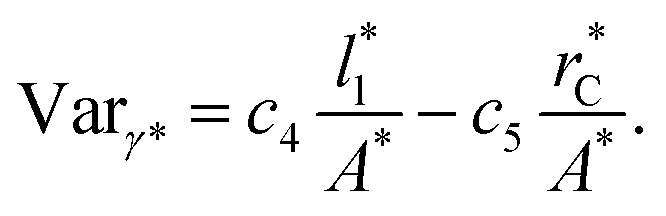
where *c*_4_ and *c*_5_ are constants. Fig. 11a shows the calculated values of Var_γ*_ as a function of 
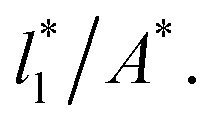
 Plotting Var_γ*_vs.
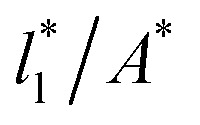
 produces linear sets of data, almost parallel, and with different intercepts to *y*-axis depending on A*, which shows significant contributions of the second term of expression (5). The slight visual differences in the profile for 
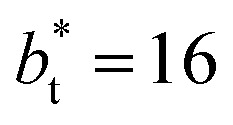
 are probably the result of the non-quadratic behavior of *σ*_γ*_ with 
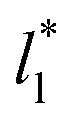
 (eqn (4)). The intercepts with *y*-axis show a linear behavior when they are plotted against the variable 1/A* (Fig. 11b) in agreement with expression (5). The simulations of Longford *et al.*^1^ using a 
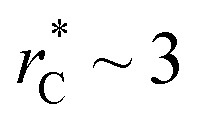
 (10 Å) show a linear behavior with almost-zero contributions of the second term of expression (5). The different behavior is clearly due to the large 
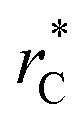
 used in this work (7.5), and predicted by the expression developed by Longford *et al.*^1^ The surface tension results of Longford *et al.*^1^ included long-range corrections to the surface tension, those corrections are based on the density profiles, but for systems under the same coexisting bulk densities and same 
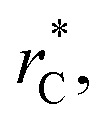
 those contributions should also be the same, therefore the observed null contributions of the second term in the work of Longford *et al.*^1^ are expected, because the dynamics of the systems, and therefore the distributions of γ* are developed basically at the 
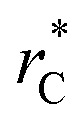
 of 3.

**Fig. 12:**
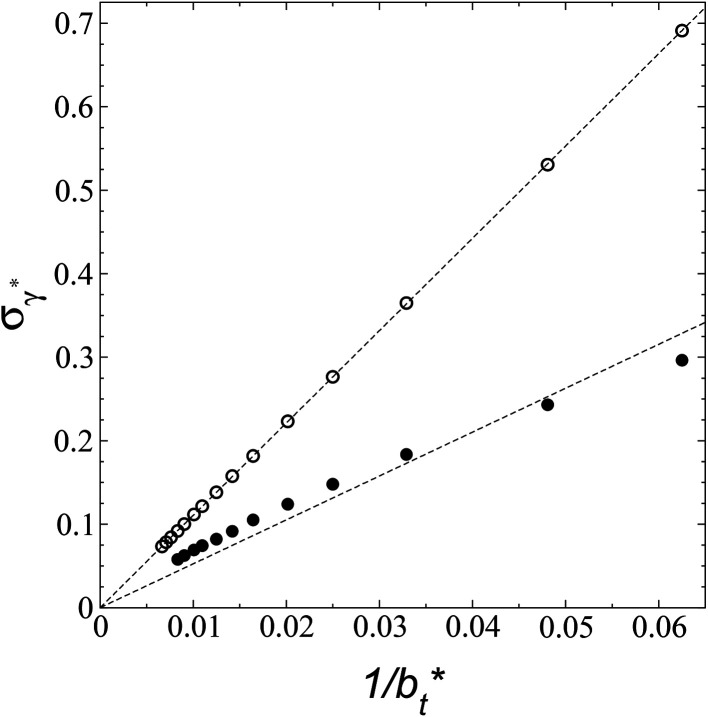
Standard deviation of the reduced surface tension as a function of the inverse of the length of the simulation cell in the tangential direction. The systems are composed of Lennard-Jones atoms at a reduced temperature of 0.72. Closed and open circles represent data for thin layers with thicknesses of 8.0 and 16.8, respectively. The discontinuous lines represent the best fits to an inversely asymptotic function (eqn (6)).

A plot of *σ*_γ*_ as a function of 
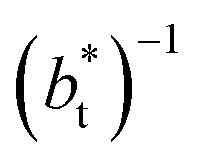
 at constant 
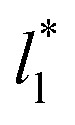
 allowed us to predict the behavior of *σ*_γ*_ at long 
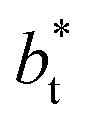
 values. Fig. 12 shows *σ*_γ*_ as a function of 
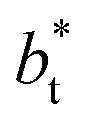
 for two thin layers with 
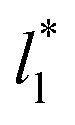
 of 8.0 and 16.8. The two sets of data show an asymptotic behavior of *σ*_γ*_ with 
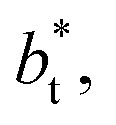
 in agreement with the expression developed by Longford *et al.*,^1^ which can be modeled as an inversely asymptotic function of the form:6
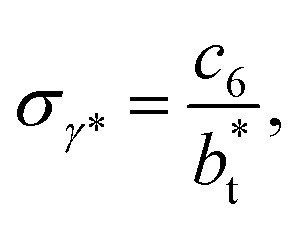
which produces values for the *c*_6_ constant for the system with 
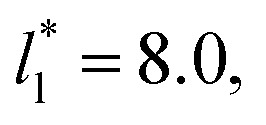
 of 5.26 (correlation coefficient of 0.9968). For the system with 
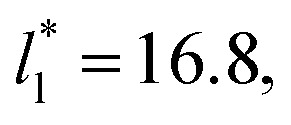
, the constant is 11.06 (0.9999). The lower agreement for the data using 
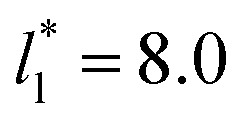
 probably is the result of the small thickness of the layers, which are closer to the critical thickness. In the hypothetical case that we wanted to measure γ* at T*= 0.72, using a very large simulation cell in the tangential direction to reduce the fluctuations of γ* to less than 1% of its total value, we could not do it with a thin layer with 
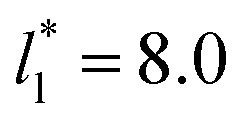
 as this value is lower than the expected critical thickness free of the size effects,^4^ and with a layer with 
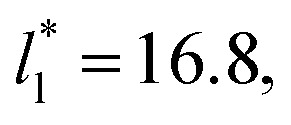
 we would need a simulation cell with 
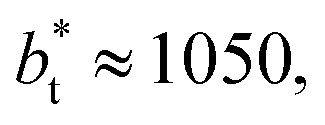
 which corresponds to a simulation cell with tangential direction dimensions at the micrometer scale (for argon or methane).

**Fig. 13:**
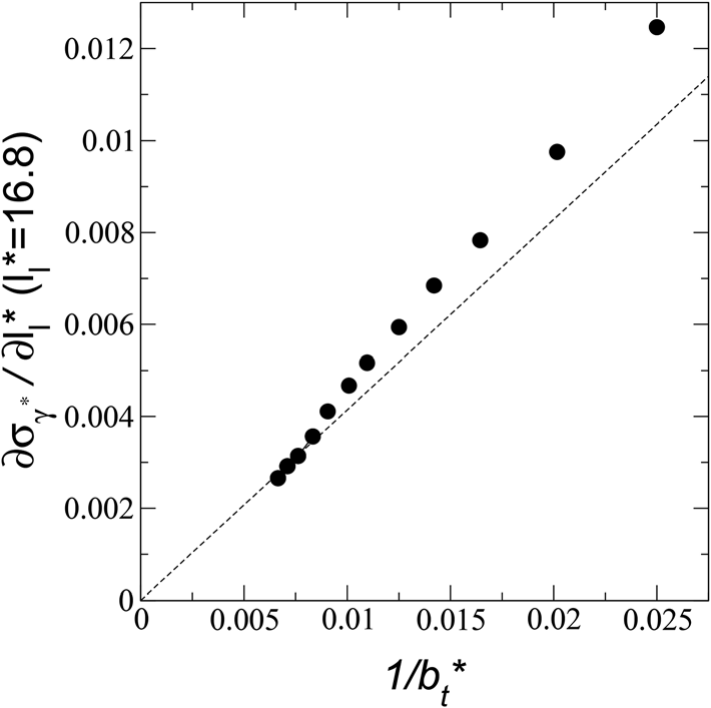
Change in the standard deviation of the reduced surface tension as a function of the inverse of the length of the simulation cell in the tangential direction. The systems are composed of Lennard-Jones atoms at a reduced temperature of 0.72 and reduced thicknesses of ≈16.8. The discontinuous line represents the best fit to an inversely asymptotic function (eqn (7)) using the 4 largest simulation cells in the tangential direction.

The *σ*_γ*_ profiles not only become more linear as 
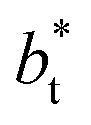
 increases, but the slope of the curves decreases (from positive values) and becomes flatter as 
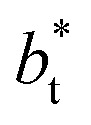
 increases. In Fig. 13, we show the calculated slope of *σ*_γ*_ with 
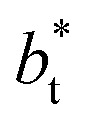
 for the systems with 
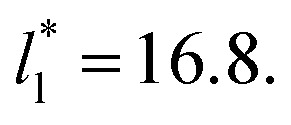
 Comparing the systems using 
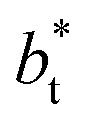
 of 40 and 150.4, the results indicate that increases in the fluctuations as large as the value of γ* are obtained when we increase the thickness of the layer by ≈80 reduced units of length at 
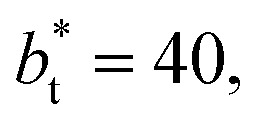
 and ≈380 reduced units of 
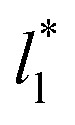
 at 
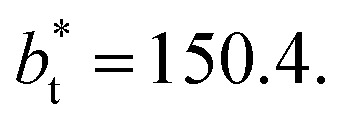
 The set of data also seems to follow an inversely asymptotic behavior, in agreement with expression (5), and the data was fitted to the corresponding expression:7
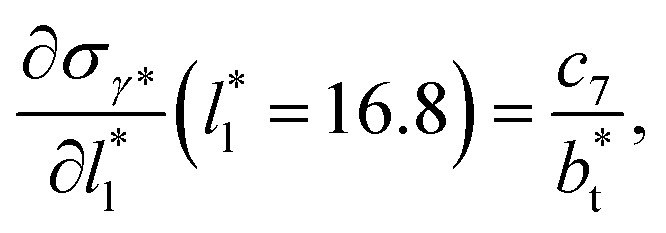
which produces values for the fitting coefficient *c*_7_ of 4.14 × 10^−1^ using the 4 largest 
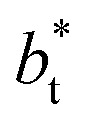
 values (correlation coefficient of 0.9980). The results for the smaller values of 
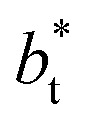
 deviates positively from the predicted behavior. This model indicates that there is a large value for 
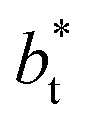
 at which *σ*_γ*_ does not grow or grows very slowly as 
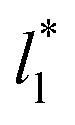
 increases.

## Conclusions

Extrapolations of data obtained from molecular dynamics simulations of the vapor/liquid equilibrium of Lennard-Jones atoms at a temperature close to the triple point, have shown that simulation cells with transverse lengths reaching μm values are needed to minimize the fluctuations (standard deviations ≤ 1%) of the surface tension, and probably other physicochemical properties, present when narrow liquid layers are simulated (composed of ≈17 monolayers). Narrower liquid layers, composed of ≈8 monolayers, will never reach such small standard deviations. Probably, liquid layers wider than 17 monolayers will require simulation cells with transverse lengths shorter than those used in this work to minimize the variance of the surface tension.

The behavior of the distributions of the surface tension, in terms of its standard deviation, shows an opposite dependence on two finite-size parameters: the length of the simulation cell in the tangential direction reduces the magnitude of the standard deviation as longer lengths are employed, while the thickness of the layer makes the standard deviation of the surface tension grow as wider layers are simulated. The dependence with the length of the layer disappears when larger lengths in the tangential direction are employed. The variance of the surface tension using a large cutoff radius follows the previously reported two-term expression with the first term dependent on the two size effects studied in this work. The difference with previous simulations using a short cutoff radius and long-range corrections is the larger contributions of the second term dependent only on the interfacial area (transverse length) of the simulation cell. The distribution of values of the surface tension using the large 
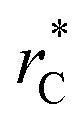
 employed in this work cannot be reproduced using small 
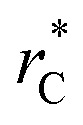
 and post-simulation long-range corrections.

The effect of the length of the simulation cell in the transverse direction is due to the introduction of artificial cohesive forces at the interfaces in both directions, resulting from the use of periodic boundary conditions. This contribution has a stronger effect when short interfacial areas are employed. When very narrow liquid layers are simulated (near the critical length), the interfaces of the system interact with each other, probably coordinating the interfacial behavior of each other. As wider lengths of the liquid layer are employed, the interfaces become less constrained, and there is no coordination between these, making them free to develop their own dynamics. For larger surface areas, the variance of the surface tension is expected to become vanishingly small as the transverse length of the simulation cell (reduced surface area) grows to very large values, but this limiting behavior is only achieved when the reduced transverse length of the simulation cell is greater than 120. For smaller transverse lengths than 120, there is a limit value in the variance of the surface tension that depends on the transverse length of the simulation cell. Given the agreement of our results with the capillary wave theory at low temperatures, we expect larger amplitude waves at higher temperatures so that larger surfaces areas (larger transverse lengths and simulation system sizes) will be required to obtain surface tension estimates with the low standard deviations of the present paper.

The size effect due to the finite size of the simulation cell in the interfacial area is clearly an artefact of the methodology employed to simulate phase equilibria under periodic conditions in the interfacial surface, but for systems confined to these interfacial-length scales, probably this size effect has a physical meaning and future work should elucidate its influence. The size effect due to the thickness of the layer is only important for narrow layers in the scale of the nanometer, beyond that its influence decreases until they disappear at larger thicknesses layers. In the future, we also plan to assess the effects of additives and multicomponent equilibria to study the dependence of finite size effects on the distributions of their interfacial properties.

## Conflicts of interest

There are no conflicts of interest to declare.

## Supplementary Material

RA-009-C9RA07058C-s001
