# Supplementary material for: Reducing uncertainty in simulation estimates of the surface tension through a two-scale finite-size analysis: thicker is better
Source: RSC Adv. 2019 Nov 4;9(61):35803–12. doi: 10.1039/c9ra07058c (PMC9074429; doi:10.1039/c9ra07058c)
Supplement: RA-009-C9RA07058C-s001 [file RA-009-C9RA07058C-s001.pdf]

Electronic Supplementary Material (ESI) for RSC Advances.
This journal is © The Royal Society of Chemistry 2019

Reducing Uncertainty in Simulation Estimates of the Surface Tension Through a Two-Scale Finite-Size Analysis: Thicker is Better

José L. Rivera[a,*] and Jack F. Douglas[b]

[a]Laboratorio de Modelamiento y Simulación Molecular, Universidad Michoacana de San Nicolás de Hidalgo, Morelia, Michoacán 58000, México

[b]Materials Science and Engineering Division, National Institute of Standards and Technology, Gaithersburg, Maryland 20899, USA

Supplemental Information

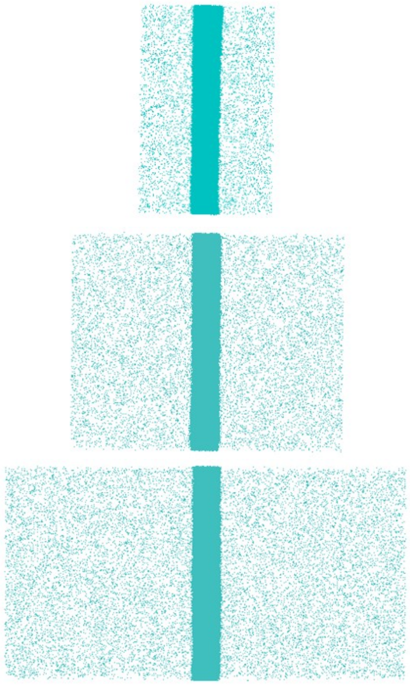

Figure S1. Orthogonal projection of a snapshot of the central cells used to simulate large bulk vapor in thermodynamic equilibrium with a liquid layer at 11 ns of simulation time. The length of the simulation cells in the homogeneous directions (parallel to the interface) are 150.4 in reduced units. The simulation cells have dimensions in the inhomogeneous directions of 96 (top), 192 (central), and 288 (bottom). Each sphere represents a Lennard-Jones atom at a reduced temperature of 0.72.

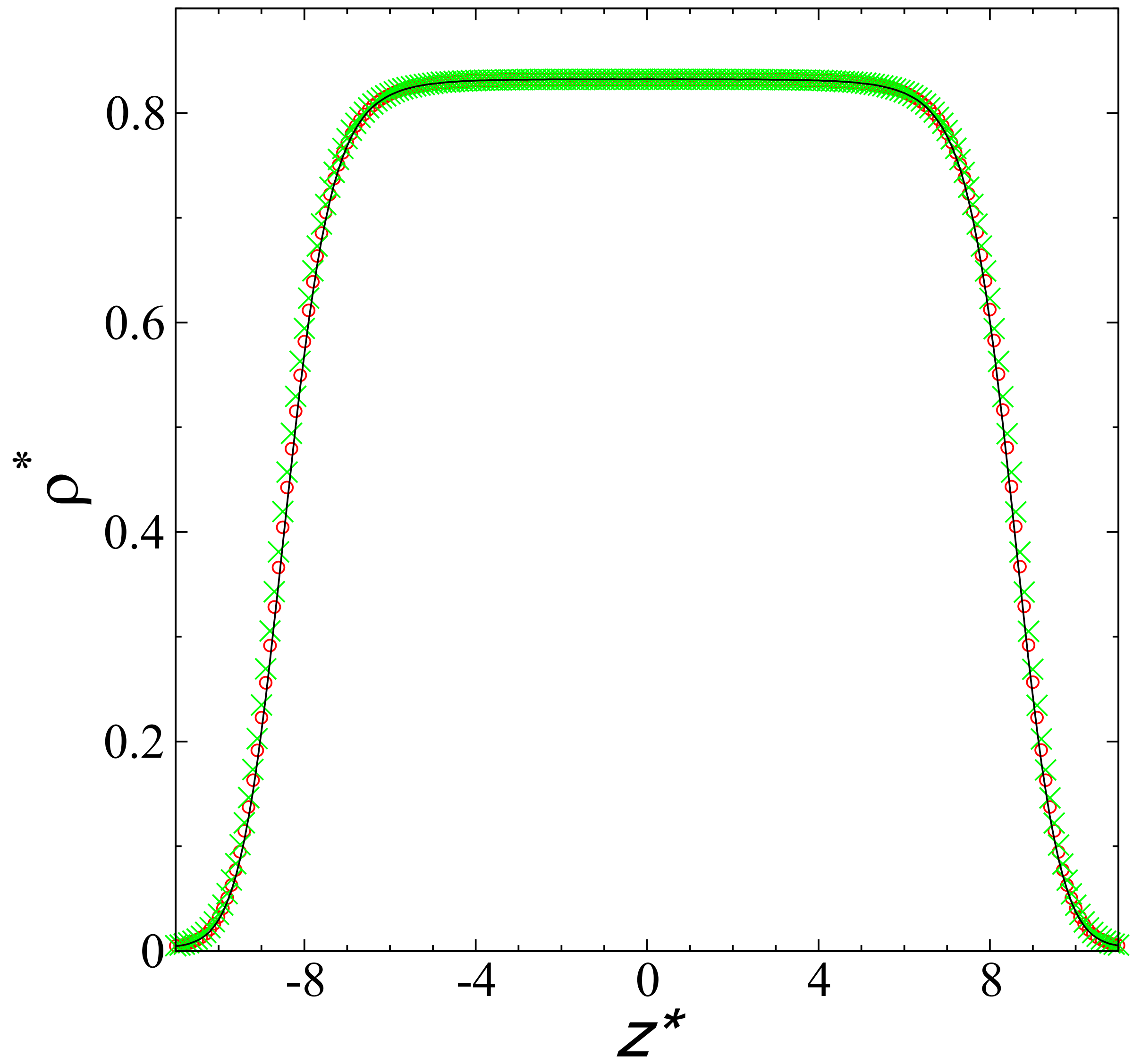

Figure S2. Reduced density profiles as a function of the position in the inhomogeneous direction of the simulation cell. The profiles represent average profiles over 10 ns. The systems are composed of Lennard-Jones atoms at a reduced temperature of 0.72 and reduced thicknesses of ≈ 16.8. The black line, the red circles, and the green crosses correspond to simulated systems using reduced lengths of the simulation box in the homogeneous direction of 96, 192 and 288, respectively. The lengths of the simulation box in the tangential direction is 150.4.

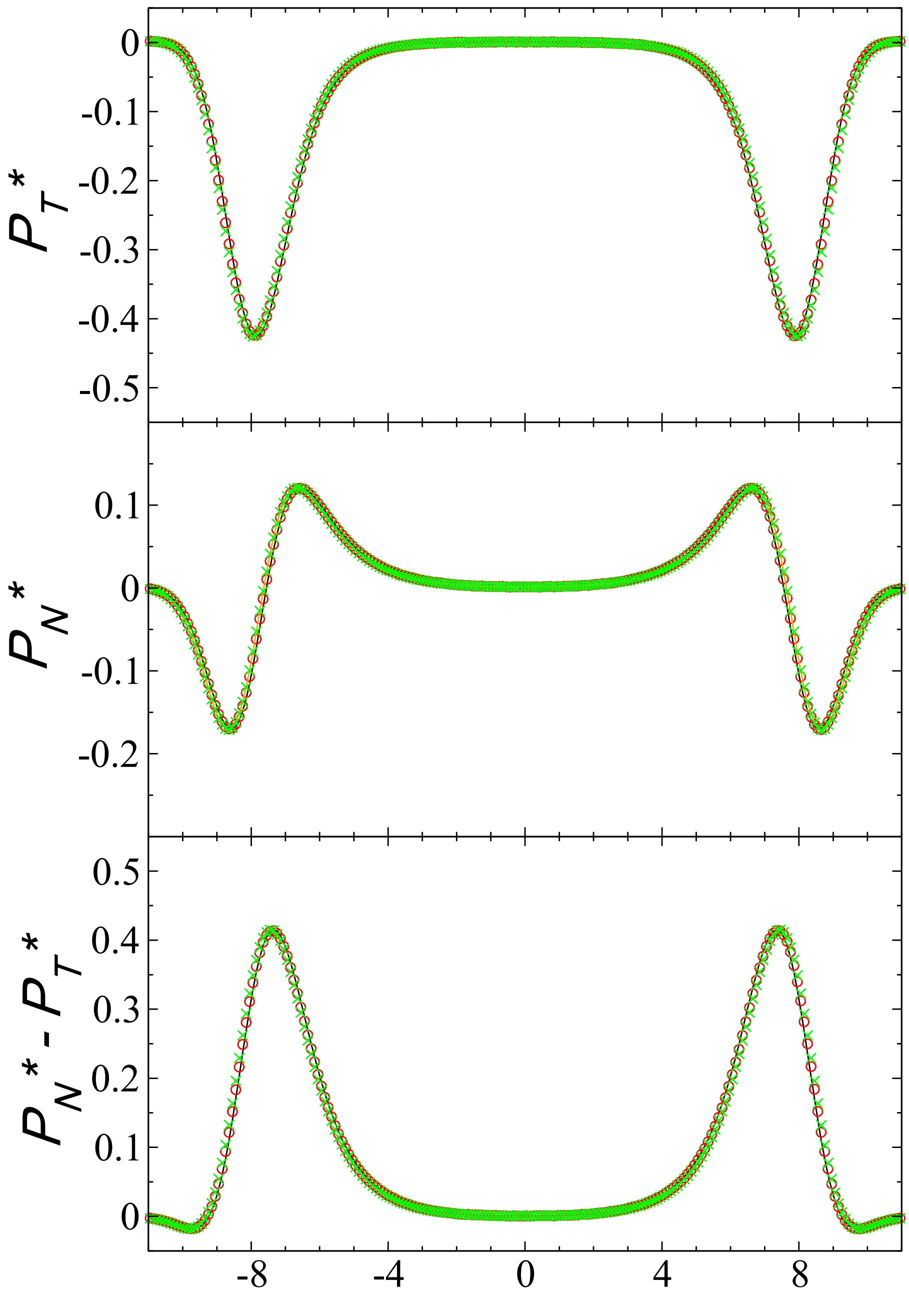

Figure S3. Average tangential (top) and normal (center) pressure profiles, and the differences between them (bottom). The systems are composed of Lennard-Jones atoms at a reduced temperature of 0.72 and reduced thicknesses of ≈ 16.8. The black line, the red circles, and the green crosses correspond to simulated systems using reduced lengths of the simulation box in the homogeneous direction of 96, 192 and 288, respectively. The lengths of the simulation box in the tangential direction is 150.4.
